# Dengue Virus and Autophagy

**DOI:** 10.3390/v3081332

**Published:** 2011-08-04

**Authors:** Nicholas S. Heaton, Glenn Randall

**Affiliations:** Department of Microbiology, The University of Chicago, Chicago, IL 60637, USA; E-Mail: nheaton@bsd.uchicago.edu

**Keywords:** lipid metabolism, lipophagy, lipid droplet, β-oxidation

## Abstract

Several independent groups have published that autophagy is required for optimal RNA replication of dengue virus (DENV). Initially, it was postulated that autophagosomes might play a structural role in replication complex formation. However, cryo-EM tomography of DENV replication complexes showed that DENV replicates on endoplasmic reticulum (ER) cisternae invaginations and not on classical autophagosomes. Recently, it was reported that autophagy plays an indirect role in DENV replication by modulating cellular lipid metabolism. DENV-induced autophagosomes deplete cellular triglycerides that are stored in lipid droplets, leading to increased β-oxidation and energy production. This is the first example of a virus triggering autophagy to modulate cellular physiology. In this review, we summarize these data and discuss new questions and implications for autophagy during DENV replication.

## 1. Introduction to Autophagy

Classical autophagy is a homeostatic process wherein cytoplasmic material is sequestered in double membrane vesicles and degraded [1,2]. The process of autophagy is initiated when a cell perceives a signal such as starvation or pathogen infection. These signals are integrated through mTOR to initiate a pathway autophagosome formation, which requires numerous autophagy (ATG) proteins, which are conserved from yeast to humans [3]. Although the process of autophagosome formation is not completely understood, many of the required components are known [4]. A limited description of autophagosome formation follows. For more comprehensive reviews on autophagy as a process, please see [2,3,5]. Initially, an immature autophagosome, termed a phagophore, forms from a variety of cytoplasmic membrane compartments [5]. The ATG1/ULK complex positively regulates phagophore formation, while ATG6/Beclin-1 recruits a class III phosphatidylinositol 3-kinase (VPS34) and ATG14 to generate phosphatidylinositol 3-phosphate and nucleate the phagophore [6]. Two ubiquitin conjugating systems are involved in the elongation of the phagophore into an isolation membrane. First, ATG5 is conjugated to ATG12, which then associates with ATG16L1 to form the pre-autophagosomal structure. Second, ATG8/LC3 is cleaved by ATG4 to form LC3-I and subsequently becomes conjugated to phosphatidylethanolamine to form LC3-II and specifically associate with autophagosomal membranes [7]. Once the isolation membrane recognizes its cargo, it then engulfs the cargo and fuses, generating an autophagosome. LC3-II plays critical roles in cargo identification and membrane fusion [8].

After a mature autophagosome has enveloped its cargo, it fuses with a lysosome to form a degradative compartment, the autolysosome. Once the contents of the autolysosome have been broken down, the components are released for use by the cell. The process of autophagy is critical for the maintenance of cellular homeostasis as well as providing a mechanism to avoid cell death during starvation conditions [3]. Outside of the context of viral infection, deregulation of autophagy can lead to various pathologies including heart disease, neurodegeneration and cancer [2].

During viral infection, autophagy can play either a pro- or anti-viral role [9]. Autophagy can act as an anti-viral component of the innate immune system, presumably by sequestering and degrading viral structures in the cells to help reduce viral replication. Autophagy can be induced by toll-like receptor (TLR) ligands, which further indicates that autophagosomes can have anti-viral functions [10–12]. In addition, autophagy can also play a role in delivering viral antigens for presentation to TLRs as has been reported for Sendai virus and vesicular stomatitis virus [13]. Autophagy can also function in the adaptive immune response. Recent studies have found that autophagy in antigen presenting cells can facilitate loading of antigen onto MHC class II molecules [14,15]; in at least one case this occurs via the fusion of autophagosomes with multi-vesicular MHC class II loading compartments [16].

Despite its antiviral functions, autophagy is subverted by some viruses to perform pro-viral roles [17]. Poliovirus, coxsackievirus B3 virus, coronaviruses, hepatitis C virus, and dengue virus are among some of the best characterized examples of viruses that activate and require some aspect of autophagy for robust viral replication [18–24]. While the exact mechanisms of how autophagy can contribute to viral replication are for the most part unclear, progress has been made in characterizing the proviral roles of autophagy. Some picornaviruses appear to use autophagosomal membranes as components of the viral replication complex [18–20]. Other viruses such as coronaviruses can utilize specific components of the autophagy machinery; in this case a non-lipidated form LC3, to help reorganize cellular membranes [21]. Hepatitis C virus also has a requirement for autophagy [22], likely for an early viral RNA translation step [23] and/or suppressing innate antiviral immunity [24].

## 2. Dengue Virus Infection Induces and Requires Autophagy

Dengue virus (DENV) is a positive-stranded RNA virus of the family *Flaviviridae.* It is composed of a group of four serotypes (DENV1-4). DENV is transmitted to vertebrate hosts via the mosquito vectors *Aedes aegypti* or *Aedes albopictus*. Infection with DENV can lead to a spectrum of clinical diseases ranging from subclinical infection to dengue fever to the most severe forms, dengue hemorrhagic fever and dengue shock syndrome [25]. Globally, there are an estimated 50–100 million infections annually, making DENV the most important arbovirus to human disease [26]. Due in part to the large impact on human health, basic research on DENV has expanded in recent years.

DENV initiates infection of a permissive cell via clathrin-mediated endocytosis and then releases its genomic RNA into the cytosol after fusing with the late endosome [27,28]. The viral RNA is translated as one open reading frame, and is subsequently cleaved by cellular and viral proteases to release three structural proteins and seven non-structural proteins. The non-structural proteins replicate the viral RNA and the structural proteins assemble with the nascent viral RNA to generate new virions [29]. During viral infection, the virus manipulates many different cellular pathways, including autophagy. DENV has been published to induce and require autophagy by four independent laboratories [30–34]. Lee *et al.* performed the initial characterization of autophagy during DENV infection in 2008 [31]. The authors showed that DENV2 infection of a hepatocyte cell line induced autophagy and that inhibiting autophagy with the drug 3-methyladenine (3MA) or siRNAs targeting autophagy gene expression compromised viral infection. They further showed that the DENV induced autophagosomes co-localized with LAMP1, a marker of lysosomal fusion.

This work was expanded upon the next year by Panyasrivanit *et al.* [33]. It was again shown that DENV2 infection of hepatocytes induced and required autophagy via immunofluorescence assays and drug inhibition. It was also shown that a proportion of DENV nonstructural protein 1 (NS1) protein co-localized with autophagosomes as well as LAMP1 and the ribosomal protein L28. The authors also showed that an endosomal marker (M6P-R) co-localized with autophagosomes, indicating that some autophagosomes may fuse with endosomes to form organelles called amphisomes. Since DENV replicates on virally induced characteristic double membrane vesicles (DMVs), and autophagosomes are DMVs, the authors hypothesized that DENV might replicate on amphisomes and thus link virus entry and replication. The authors also showed that inhibiting lysosomal fusion with autophagosomes increased viral replication, indicating a role for immature autophagosomes during DENV replication.

Soon after this publication, work from the same lab (Khakpoor *et al.*) examined the role of autophagy in DENV3 infection [32]. Similar to DENV2, DENV3 infection also induced and required autophagy. LAMP1 was observed to co-localize with autophagosomes, but in contrast to the previous DENV2 study, treatment with a lysosomal fusion inhibitor decreased DENV3 replication. This indicated a role for mature autolysosomes in DENV3 infection. The mechanism for how autolysosomes could contribute to viral replication remained unclear.

## 3. The Structure of Dengue Virus Replication Complexes

Following these initial characterizations of DENV-induced autophagy, an electron tomography study was performed by Welsch *et al.* which showed the 3D structure of DENV2 replication complexes in hepatocytes [35]. While traditional thin section EM appears to show virally induced replication complexes to be a cluster of independent double membrane vesicles [36], the 3D reconstruction clearly showed that these vesicles were actually contiguous invaginations of the ER. Complementary immuno-EM studies demonstrated that the viral replicase proteins are present on the ER invaginations as well as double-stranded RNA, the viral replication intermediate [35].

Interestingly, the ER invaginations that contain the replication complex were physically linked to ER membrane compartments that contained capsids [28]. A model was proposed wherein DENV RNA is transported through a neck that links the replication complex compartment to sites of capsid assembly. Following assembly, the capsid would bud into the ER to acquire its envelope. Thus, this study clearly ruled out the hypothesis that DENV was replicating on classical autophagosomes; however, there was still a very clear requirement for autophagy during viral replication. This indicated that autophagy might be playing an indirect role to enhance viral replication.

## 4. DENV Induces a Selective Autophagy that Stimulates Lipid Metabolism

In addition to bulk macroautophagy, which is relatively non-specific, different types of selective autophagy exist that target specific organelles (reviewed in [37]). The hypothesized role of selective autophagy is that the cell frequently needs to initiate a physiological response that appropriately addresses a specific stress. Relevant to DENV infection, a type of selective autophagy termed lipophagy was described, wherein autophagosomes can target cellular stores of lipids known as lipid droplets (LDs) to generate energy for the cell [38]. Heaton *et al.* performed a limited siRNA screen to identify cellular co-factors of DENV2 replication in hepatocytes, which identified, among others, a gene involved in the induction of autophagy [30]. In subsequent work, the authors reproduced the published results that DENV induces and requires autophagy for robust viral replication [39]. The initial observations were expanded upon by showing that DENV induced autophagosomes not only acquire LAMP1, but complete their maturation and become autolysosomes [39]. These autophagosomes did not co-localize with markers of the viral replication complex, suggesting that they may play an indirect, non-structural role in DENV replication.

The DENV-induced autophagosomes did, however, significantly co-localize with lipid droplets. Lipid droplet volume decreased during DENV infection in an autophagy-dependent manner, as did cellular triglycerides, a major component of lipid droplets. DENV-induced autophagy stimulated the delivery of lipids to lysosomal compartments, resulting in the release of free fatty acids, which undergo β-oxidation in the mitochondria to generate ATP. This produces a metabolically favorable environment for viral replication. Importantly, the authors showed that the defect in viral replication caused by inhibition of autophagy could be completely complemented by adding exogenous free fatty acids. This complementation of defective autophagy by free fatty acids required β-oxidation. Thus, despite the many roles of autophagy in regulating cellular homeostasis, its regulation of lipid metabolism is a major contributor for robust DENV replication [39].

Many viruses, including cytomegalovirus, HCV, and DENV alter cellular metabolism to promote their replication (reviewed in [40]). The induction of lipophagy is a novel mechanism by which viruses can manipulate the metabolic state of the infected cell. It is also a very different interaction with autophagy than has been proposed with other viral infections. Many viruses appear to induce a bulk autophagy and then inhibit its progression at various steps to prevent anti-viral functions (reviewed in [17]). Alternatively, DENV infection induces a selective autophagy that is preferentially targeted to lipid droplets, which leads to changes in cellular metabolism. In addition to modifying cellular metabolism, it is possible that this serves a secondary function in immune evasion. The targeting of autophagosomes to lipid droplets may also divert autophagosomes from processing viral antigens for antigen presentation as an immune evasion strategy.

The mechanism by which DENV induces autophagy is unclear. A recent report showed that NS4A expression can induce autophagosome formation during DENV infection and help infected cells avoid apoptosis in renal epithelial cells and thus, contribute to prolonged viral replication [34]. The unfolded protein response (UPR)/autophagy pathways have been shown to modulate the DENV pathogen-associated molecular pattern (PAMP) RNA-induced innate immune response [24], suggesting that autophagy may promote DENV replication through repressing innate immunity. More work, however, is required to show whether the proposed viral triggers of autophagy reproduce all cellular signals and phenotypes that accompany autophagy induction in DENV-infected cells.

## 5. Conclusions and Future Directions

**Figure 1.**
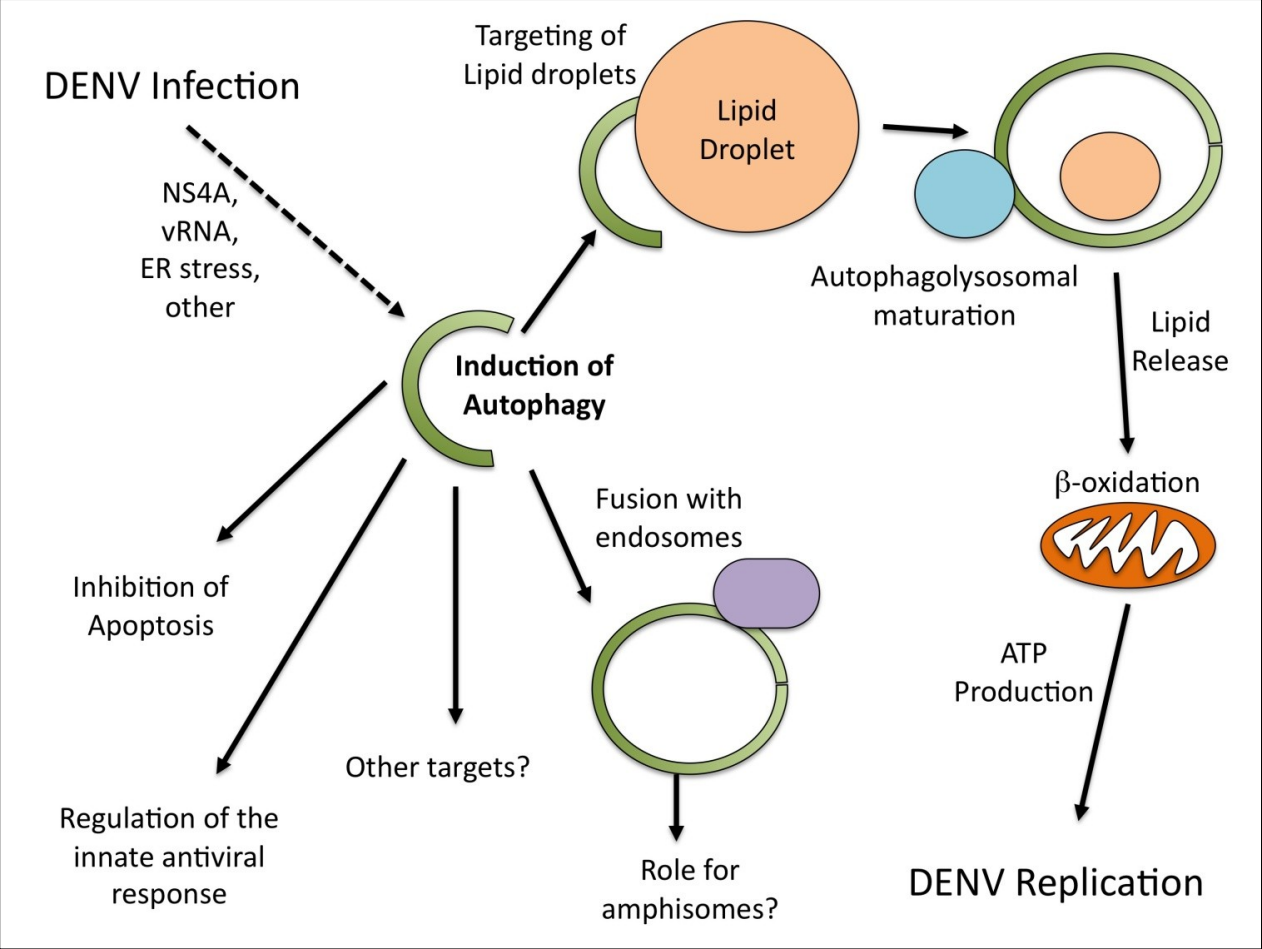
Roles for autophagy during dengue virus (DENV) infection.

Given the many functions of autophagy in the cell, it is perhaps not surprising that different studies have identified multiple possible roles for autophagy in DENV infection (Figure 1). While it is clear that DENV replication does not occur on discreet, classical autophagosomes but rather the ER, there are possible explanations as to why DENV proteins are sometimes observed on membranes positives for autophagosomal markers. Perhaps the components of the autophagosomal machinery are involved in the ER membrane reorganization. This has been shown to be the case for the coronavirus mouse hepatitis virus [21].

Additionally, it is possible that DENV may have different interactions with autophagy, dependent on the cell type. The majority of studies with DENV and autophagy have focused on non-phagocytic cells, including hepatocytes and epithelial kidney cells. The impact of autophagy on DENV replication also needs to be characterized in phagocytic cells. While the liver is an *in vivo* target during DENV infection, it will be important to repeat these experiments in monocytes, which are thought to be a primary target during infection. Further, the majority of this work has been done with DENV2, with only one report examining DENV3. It will be important to determine which of the interactions between DENV and autophagy are conserved between serotypes.

Autophagy responds to various stress stimuli to maintain cellular homeostasis. Since viral infections frequently induce stress, it was initially assumed that autophagy induction might be a byproduct of the infection. In this model, autophagy would be generically triggered and then subsequently inhibited by a viral protein prior to maturation into its degradative form. The demonstration that DENV can induce at least one form of selective autophagy that goes to completion to modify cellular metabolism suggests that it triggers either the induction or the marking of lipid droplets as cargo in a very specific way. An important question is how the stimulation of ATP production by DENV-induced autophagy benefits replication. The answer that “energy is good” is not wholly satisfying. ATP production might impact cellular energetics, ATP-dependent enzymatic processes required for replication such as the NS3 ATPase activity, or cellular signaling pathways that are regulated by ATP levels. The importance of viral modulation of cellular metabolism is an emerging field within virology with more questions than answers at this stage.

The cellular pathways that are modified and the viral functions responsible for lipophagy induction are not yet known. Indeed, the cellular pathway of lipophagy induction is also poorly characterized. Future studies characterizing the mechanism by which DENV induces lipophagy should enlighten our understanding of how selective autophagy is triggered. It may also produce a novel antiviral therapeutic strategy. A generic interference with autophagy is a dubious antiviral approach given the importance of autophagy to cellular survival. However, it is possible that a DENV-specific interaction with the autophagy machinery may be targeted for therapeutic intervention. Inhibition of the DENV-autophagy interaction stimulating lipophagy would limit cellular metabolic changes and inhibit DENV replication. It may also enhance immune recognition of DENV antigens, since autophagosomes would no longer be diverted to lipid droplets.

In sum, there have been many proposals for the role of autophagy during DENV infection. One role is the modulation of the cellular metabolic state, however, this is not mutually exclusive with additional pro-viral roles (such as the inhibition of apoptosis). Future work will help to further characterize the role(s) and relative contributions of autophagy to the various aspects of DENV replication.

## Figures and Tables

**Table 1.** Summary of the studies relating autophagy to dengue virus (DENV) infection.

**Study**	**DENV Serotype**	**Cell Lines Used**	**Major Conclusions**
Lee *et al*., 2008 [31]	DENV2	Huh7	DENV induces autophagy DENV induced autophagosomes acquire LAMP1 3MA treatment reduces viral replication Silencing ATG5 inhibits viral replication
Panyasrivanit *et al.*, 2009 [33]	DENV2	HepG2	DENV induces autophagy DENV induced autophagosomes acquire LAMP1 3MA treatment reduces viral replication DENV NS1 and dsRNA can be detected on LC3 positive structures Inhibiting autophagosome/lysosome fusion increases virus yield
Khakpoor *et al.*, 2009 [32]	DENV3	HepG2	DENV induces autophagy DENV induced autophagosomes acquire LAMP1 3MA treatment reduces viral replication DENV NS1 and dsRNA can be detected on LC3 positive structures Inhibiting autophagosome/lysosome fusion decreases virus yield
Heaton *et al.*, 2010a [30]	DENV2	Huh7.5	Silencing ATG6 inhibits viral replication
Heaton *et al*., 2010b [39]	DENV2	Huh7.5, BHK, Huh7, HepG2	DENV induces autophagy 3MA or ATG siRNAs reduce viral replication and virus production, but not entry or protein translation No significant co-localization between LC3 and viral replication markers DENV induced autophagosomes associate with lipid droplets DENV induced autophagosomes acquire LAMP1 and acidify DENV infection increases the delivery of lipids to autolysosomes Dengue induced autophagy leads to depletion of cellular triglycerides and stimulation of beta-oxidation The requirement of autophagy for DENV replication can be supplanted by adding exogenous fatty acids. This is dependent on beta-oxidation.
Ke *et al.*, 2011 [24]	DENV2	Huh7	Autophagy can reduce the activation of the interferon beta promoter stimulated by DENV 3′-UTR RNA
McLean *et al*., 2011 [34]	DENV2	MDCK, MEF, 293T, HeLa, Vero	DENV infected cells are resistant to apoptosis by exogenous stimuli. DENV induces autophagy 3MA treatment inhibits viral replication Inhibition of autophagy prevents DENV mediated resistance to apoptosis Over-expression of NS4A alone prevents apoptosis NS4A mediated protection from apoptosis is dependent upon autophagy
